# RETRACTION: MeCP2 Drives Hepatocellular Carcinoma Progression Via Enforcing HOXD3 Promoter Methylation and Expression Through the HB‐EGF/EGFR Pathway

**DOI:** 10.1002/1878-0261.13681

**Published:** 2024-06-05

## Abstract

**RETRACTION:** L. Wang, Y. Gao, D. Tong, X. Wang, C. Guo, B. Guo, Y. Yang, L. Zhao, J. Zhang, J. Yang, Y. Qin, L. Liu, and C. Huang, “MeCP2 Drives Hepatocellular Carcinoma Progression Via Enforcing HOXD3 Promoter Methylation and Expression Through the HB‐EGF/EGFR Pathway,” *Molecular Oncology* 15, no. 11 (2021): 3147‐3163, https://doi.org/10.1002/1878-0261.13019.

The above article, published online on 24 May 2021 in Wiley Online Library (wileyonlinelibrary.com), has been retracted by agreement between the journal Editor‐in‐Chief, Kevin Ryan; FEBS Press; and John Wiley and Sons Ltd. The retraction has been agreed upon following an investigation into concerns raised by a third party regarding an image duplication between Figures 2C (MeCP2 panel) and 4G (left panel). The investigation also revealed inconsistencies in the gel blots of Figures 2J and 3N. The partial raw data shared by the authors was deemed insufficient to address these concerns. Therefore, the editors have lost confidence in the presented data, consider the conclusions substantially compromised and are retracting the paper. The authors were informed of the decision to retract but did not agree to the retraction or the retraction wording.

**RETRACTION:** L. Wang, Y. Gao, D. Tong, X. Wang, C. Guo, B. Guo, Y. Yang, L. Zhao, J. Zhang, J. Yang, Y. Qin, L. Liu, and C. Huang, “MeCP2 Drives Hepatocellular Carcinoma Progression Via Enforcing HOXD3 Promoter Methylation and Expression Through the HB‐EGF/EGFR Pathway,” *Molecular Oncology* 15, no. 11 (2021): 3147‐3163, https://doi.org/10.1002/1878-0261.13019.

The above article, published online on 24 May 2021 in Wiley Online Library (wileyonlinelibrary.com), has been retracted by agreement between the journal Editor‐in‐Chief, Kevin Ryan; FEBS Press; and John Wiley and Sons Ltd. The retraction has been agreed upon following an investigation into concerns raised by a third party regarding an image duplication between Figures 2C (MeCP2 panel) and 4G (left panel). The investigation also revealed inconsistencies in the gel blots of Figures 2J and 3N. The partial raw data shared by the authors was deemed insufficient to address these concerns. Therefore, the editors have lost confidence in the presented data, consider the conclusions substantially compromised and are retracting the paper. The authors were informed of the decision to retract but did not agree to the retraction or the retraction wording.

